# Perseverative Thinking Questionnaire (PTQ): French Validation of a Transdiagnostic Measure of Repetitive Negative Thinking

**DOI:** 10.3389/fpsyg.2017.02159

**Published:** 2017-12-13

**Authors:** Faustine Devynck, Monika Kornacka, Celine Baeyens, Éric Serra, Jérémy Fonseca das Neves, Bulle Gaudrat, Caroline Delille, Pierre Taquet, Olga Depraete, Philippe Tison, Fabienne Sgard, Amélie Rousseau, Lucia Romo

**Affiliations:** ^1^Univ. Lille, EA 4072 – PSITEC – Psychologie: Interactions Temps Émotions Cognition, Lille, France; ^2^Interdisciplinary Center for Applied Cognitive Studies, SWPS University of Social Sciences and Humanities, Warsaw, Poland; ^3^Univ. Grenoble Alpes, LIP/PC2S, Grenoble, France; ^4^Pain Unit, Centre d’Etude et de Traitement de la Douleur, Centre Hospitalier Universitaire Amiens, Amiens, France; ^5^Department of Nutrition, Centre Hospitalier d’Arras, Arras, France; ^6^Clinique Parc Monceau, Groupe Ramsay Général de Santé, Lille, France; ^7^Groupe Hospitalier Seclin Carvin, Seclin, France; ^8^Centre Hospitalier Regional et Universitaire de Lille, Lille, France; ^9^Clinique de Villeneuve d’Ascq, Villeneuve d’Ascq, France; ^10^Centre Hospitalier de Saint-Amand-les-Eaux, Saint-Amand-les-Eaux, France; ^11^Department of Psychology, University Paris Nanterre, Nanterre, France; ^12^Unité Inserm U894 CNP, Paris, France

**Keywords:** repetitive negative thinking, transdiagnostic process, rumination, worry, questionnaire, validation

## Abstract

Repetitive negative thinking (RNT) is a transdiagnostic process involved in the onset and maintenance of many psychological disorders. The Perseverative Thinking Questionnaire (Ehring et al., 2011) is a content-independent scale composed of 15 items that assesses RNT from a transdiagnostic perspective in both clinical and general populations. The aim of the current research was to translate and validate the French version of the PTQ through two studies (total *N* = 1016) following the steps for the trans-cultural validation of psychometric instruments (Hambleton et al., 2006). An exploratory factor analysis conducted on a first community sample revealed a latent structure composed of 10 items distributed on one common factor, labeled RNT, and three subfactors that evaluated the repetitive characteristic of RNT, the intrusiveness of RNT and the effect of RNT on mental resources. This factorial structure was confirmed in two confirmatory factor analyses in community and clinical samples. Scale score reliability indices were good and confirmed the validity of the instrument. The French version of the PTQ is a good content-independent instrument to assess RNT in general and clinical populations of French speakers.

## Introduction

Repetitive negative thinking (RNT) is defined as excessive and repetitive thinking about negative topics that is experienced as difficult to control (Ehring and Watkins, 2008). This cognitive process is an emotion regulation strategy involved in the development, maintenance and recurrence of a large number of disorders, such as anxiety disorders (Ehring and Ehlers, 2014; Arditte et al., 2016), depression (Nolen-Hoeksema et al., 2008; Watkins, 2008), alcohol use disorders (Caselli et al., 2013), eating disorders (Nolen-Hoeksema et al., 2007), and pain disorders (Edwards et al., 2011). Based on this growing body of studies, RNT is considered a transdiagnostic process (Ehring and Watkins, 2008; Nolen-Hoeksema and Watkins, 2011), or “a process underlying multiple, usually comorbid, psychopathologies” (Nolen-Hoeksema and Watkins, 2011, p. 589).

The transdiagnostic perspective leads to a better understanding of high comorbidity and similarities between diagnoses by focusing on common processes that causally contribute to psychopathological symptoms (Mansell et al., 2009; Watkins, 2015). For example, the most frequently studied forms of RNT are rumination and worry. Rumination refers to a response to a sad mood involving repetitive thoughts that focus on one’s negative emotional state and the possible causes and consequences of these negative states (Nolen-Hoeksema et al., 2008). Rumination has mainly been examined in relation to depression (Nolen-Hoeksema and Morrow, 1991; Nolen-Hoeksema et al., 2008; Watkins and Nolen-Hoeksema, 2014). Worry, the central characteristic of General Anxiety Disorder (GAD), is defined as “repetitive thoughts and images charged with negative affect relatively uncontrollable which lead to an attempt to engage in mental problem solving for which the outcome is uncertain but contains the possibility of one or more negative outcomes” (Borkovec et al., 1983, p. 9). Rumination and worry usually correlate (Watkins et al., 2005) and present more similarities than differences. The main difference between rumination and worry is their temporal orientation; rumination relates to past losses, whereas worries involve future threats (Nolen-Hoeksema and Watkins, 2011; Watkins, 2015). These shared characteristics lead us to consider rumination and worry as a transdiagnostic process involved in the onset and maintenance of disorders that are not limited to depression and GAD (Ehring and Watkins, 2008).

Researchers have traditionally used the Ruminative Response Scale-Reconsidered (Treynor et al., 2003), to evaluate rumination in response to depressed mood and the Penn State Worry Questionnaire (Meyer et al., 1990) to assess the frequency and intensity of worries and cognitive intrusions. These standard measures are based on specific-disorder definitions of rumination and worry and do not measure RNT independently of the content. To address these limitations, Ehring et al. (2011) developed the Perseverative Thinking Questionnaire (PTQ). This content-independent measure of RNT was based on the following definition: “Repetitive negative thinking as relevant to emotional problems is a style of thinking about one’s problem (current, past or future) or negative experiences (past or anticipated) that shows three key characteristics: (1a) the thinking is repetitive, (1b) it is at least partly intrusive, and (1c) it is difficult to disengage from. Two additional features of RNT are that (2) individuals perceive it as unproductive and (3) it captures mental resources” (Ehring et al., 2011, p. 226). This working definition based on characteristics common to rumination and worry led to the development of the PTQ to assess RNT from a transdiagnostic perspective.

Respondents to the PTQ are asked to describe how they typically think about negative experiences or problems and to rate on a 5-point Likert scale from 0 (never) to 4 (almost always) the extent to which each statement applies to them when they think about negative experiences or problems. The PTQ is a 15-item self-report questionnaire. It was translated and validated with good psychometric properties from its original German version (Ehring et al., 2011) into English (Ehring et al., 2011), Dutch (Ehring et al., 2012), Portuguese (Chaves et al., 2013) and Polish (Kornacka et al., 2016). The English (Ehring et al., 2011), Dutch (Ehring et al., 2012) and Polish (Kornacka et al., 2016) versions of the PTQ demonstrated the same factorial structure as the original German version (Ehring et al., 2011): one higher-order factor labelled “Repetitive Negative Thinking” and three lower-order factors. The first one is related to the key features of RNT: the repetitiveness of RNT (e.g., item 1: “The same thoughts keep going through my mind again and again”), the intrusiveness of RNT (e.g., item 7: “Thoughts come to my mind without me wanting them to”) and the difficulty of disengaging aspect of RNT (e.g., item 3: “I can’t stop dwelling on them”). The second lower-order factor represents the perceived unproductiveness of RNT (e.g., item 4: “I think about many problems without solving any of them”), and the last one is labeled RNT capturing mental resources (e.g., item 5: “I can’t do anything else while thinking about my problems”). The validation study of the Portuguese version of the PTQ (Chaves et al., 2013) revealed a different factorial structure with only two factors, “Repetitive Thought” and “Cognitive Interference and Unproductiveness.” All these versions of the PTQ demonstrated good psychometric properties (Ehring et al., 2011, 2012; Chaves et al., 2013; Kornacka et al., 2016).

The French language is spoken by more than two hundred and seventy-four million people worldwide among twenty-nine countries (French Language Observatory, 2014). As expressed by Ziegler and Bensch (2013, p. 81): “In order to be able to compare research findings from different countries and in different languages, it is important to ensure the comparability of the assessment methods used.” A French version of the PTQ would allow French-speaking researchers and therapists to assess this transdiagnostic process. The aim of the current article was to validate a French version of the PTQ among clinical and non-clinical samples. The first study was conducted to explore the psychometric properties of the French version of the PTQ in two independent community samples of French speakers using exploratory and confirmatory factor analyses. The second study explored the factorial analysis, reliability and validity of the French version of the PTQ in a clinical sample.

## Study 1

### Method

#### French Adaptation of the Scale

The PTQ was first translated into French following the steps for the *trans*-cultural validation of psychometric instruments (Hambleton et al., 2006). First, items from the English version (**Appendix, Table A**) were translated into French by two bilingual experts and then back-translated into English by three other bilingual experts. Two independent judges evaluated the accuracy of the translation, the conformity of the retranslated English version with the original English version and the linguistic precision of the French items. Only item 8 had a problematic back-translation and was appropriately amended. The French version of the scale is provided in **Appendix, Table B**.

#### Participants

Participants (*N* = 467) of the first community sample were non-clinical volunteers recruited by advertising through e-mail and through social (e.g., Facebook) and research networking websites. All participants had to be fluent in French. After removing participants with missing data and multivariate outliers (Fields, 2000), the final sample was composed of 364 participants (Female = 250), aged from 18 to 64 (*M* = 26.62, *SD* = 7.28). Participants in the second community sample (*N* = 473) were recruited in the same way as the first sample. Participants with missing data and multivariate outliers were removed (Fields, 2000). The participants of the final sample (*N* = 361; female = 250) were aged 18–64 (age: *M* = 26.68, *SD* = 8.23). In both samples, the questionnaires were completed on a web-based secured and encrypted survey (i.e., Survey Monkey). No personal data allowing personal identification was recorded. All participants gave written informed consent. The study protocol was conducted according to the recommendations of the American Psychological Association and the 1964 Declaration of Helsinki.

#### Measures

##### The Perseverative Thinking Questionnaire

The Perseverative Thinking Questionnaire (PTQ; Ehring et al., 2011) is composed of 15 items evaluating (1) the core characteristics of RNT, that is, the repetitiveness of RNT (items 1, 6, and 11), the intrusiveness of RNT (items 2, 7, and 12), and the difficulty of disengaging (items 3, 8, and 13), (2) the perceived unproductiveness of RNT (items 4, 9, and 14), and (3) RNT capturing mental resources (items 5, 10, and 15). The participants responded to each item using a 5-point Likert scale from 0 (never) to 4 (almost always). A higher score on each dimension reflects a high level of the assumed process characteristic of the RNT considered. Validation studies reported good internal consistency. In the original German-language version, internal consistency was excellent in all three samples (Sample 1: α = 0.95; Sample 2: α = 0.94; Sample 3: α = 0.95). Moreover, internal consistency was good in all factors (Factor 1: α = 0.92–0.94; Factor 2: α = 0.77–0.87; Factor 2: α = 0.82–0.90) (Ehring et al., 2011). Excellent internal consistencies were also found for the English-language version of the PTQ (the PTQ total score: α = 0.95; Factor 1: α = 0.94; Factor 2: α = 0.83; Factor 3: α = 0.86) (Ehring et al., 2011). Internal consistency was excellent for the total scale of the Dutch-language version of the PTQ in both Belgian and Dutch samples (Dutch sample: α = 0.94; Belgium sample: α = 0.93) (Ehring et al., 2012). The Portuguese version of the PTQ also demonstrated excellent internal consistency (the total score α = 0.93; Factor 1: α = 0.90; Factor 2: α = 0.87) (Chaves et al., 2013). Finally, the internal consistency of the Polish-language version of the PTQ has been described as low but adequate (α = 0.64–0.92) (Kornacka et al., 2016).

##### The Ruminative Response Scale-Reconsidered

The Ruminative Response Scale-Reconsidered (RRS-R; Treynor et al., 2003; Baeyens et al., in preparation for the French translation) was used to establish convergent validity. This scale allows for the distinction of a “reflection” factor (e.g., item 3: “Go someplace alone to think about your feelings”) and a “brooding” factor (e.g., item 9: “Think: What am I doing to deserve this?”). The participants responded using a 4-point Likert scale ranging from 1 (*almost never*) to 4 (*almost every time*). A validation study reported an acceptable level of internal consistency (for brooding, α = 0.73; for reflection, α = 0.73) and a positive correlation with depression symptoms in the general population (Baeyens et al., in preparation). In the present study, internal consistencies were acceptable for both the total score of RRS and the reflection factor of rumination but slightly low for both the brooding factor (the RRS total score, α = 0.75; brooding, α = 0.66; reflection, α = 0.70) (criteria defined by Nunnally, 1978).

##### The Penn State Worry Questionnaire

The Penn State Worry Questionnaire (PSWQ; Meyer et al., 1990; Gosselin et al., 2001 for the French validation) is a 16 items self-reported questionnaire. It evaluates the frequency and intensity of worries. Participants answered on a Likert scale ranging from 1 (*not characteristic at all*) to 5 (*extremely characteristic*) (e.g., item 2: “My worries overwhelm me”). Validation studies reported excellent validity and consistency properties in general and clinical populations with general anxiety disorder (Gosselin et al., 2001). In the present study, internal consistency for the PSWQ was high (α = 0.92).

##### The State Trait Anxiety Inventory-Trait

The State Trait Anxiety Inventory-Trait (STAI-YB; Spielberger, 1989; Gauthier and Bouchard, 1993 for the French translation) is a 20-item self-report questionnaire assessing recurrent anxiety. Participants answered on 4-point Likert scale from 1 (*never*) to 4 (*always*). Internal consistency for the STAI-YB was very high (α = 0.93).

##### The Center for Epidemiologic Studies Depression Scale Revised

The Center for Epidemiologic Studies Depression Scale Revised (CESD-R; Eaton et al., 2004 for the French translation) is a 20-item self-report questionnaire assessing depression levels over the last week. The participants answered on 4-point Likert scale from 0 (never, rarely, at least 1 day) to 3 (frequently, all the time, 5–7 days). The CESD-R was added in the second community sample study. Internal consistency for the CESD-R was good (α = 0.75).

#### Data Analysis

First, an exploratory factor analysis (EFA) using SPSS statistics 20.0 (IBM Corp Released, 2011) was performed to study the factorial structure of the French version of the PTQ in the first community sample. The three application conditions of the EFA were respected: (1) items were significantly intercorrelated, (2) the sample adequacy index was excellent (KMO = 0.923) (KMO index varies between 0 and 1 and is considered excellent beyond 0.80; Kaiser, 1970), and (3) Bartlett’s test of sphericity was significant (*χ^2^* = 3310.12, *p* < 0.001) (if the test is significant beyond 0.05, we can reject the null hypothesis and consider that all the variables are not perfectly independent of each other; Hair et al., 2006). Data from our community sample (*N* = 364) were submitted to a principal axis factoring to extract factors, with Promax rotation procedure to give an oblique solution.

Second, a confirmatory factor analysis (CFA) using Amos 23 (Arbuckle, 2014) was conducted in the second community sample to compare the goodness-of-fit for four separate models following the recommendations of Rindskopf and Rose (1988). The first correlational model (model A) was composed of three correlated subfactors: (1) Repetitiveness of RNT and difficulties in disengaging (items 1, 3, 6, and 11), (2) Mental resources captured by RNT (items 5, 10, and 15), and (3) Intrusiveness of RNT (items 2, 7, and 12). The second hierarchical model (model B) was composed of one higher-order factor (RNT) and the three lower-order factors described in model A. The third bifactor model (model C) was composed of one common factor (RNT) and the three same subfactors as in model A and B. Finally, the model validated by Ehring et al. (2011) (model D) was also tested to compare the fit indices with the model of the French version. Because the data were non-normally distributed (Mardial’s test of multivariate kurtosis = 3.31, *p* < 0.001; Small’s test of multivariate normality = 67.77, *p* < 0.001) (Mardia, 1975) and the items were ordinal, robust maximum likelihood estimation was used to examine the fit of the four models. The goodness-of-fit indices considered in these studies were the normed chi-square (the chi-square on the number of degrees of freedom) which is acceptable lower than 3 (Schumacker and Lomax, 2004), the Goodness-of-Fit Index (GFI) and its corresponding adjusted version (AGFI), two absolute fit indexes for which the minimum value for model acceptance is 0.80 (Cole, 1987), the Root Mean Square Error of Approximation (RMSEA) which is considered as good when lower than 0.05 and acceptable if lower than 0.10, and the Root Mean Square Residual (RMR) which must be as low as possible and considered as acceptable when lower than 0.05 (Cole, 1987), the Comparative Fit Index (CFI) comparing the model of interest with alternatives which have to exceed 0.90 (Hu and Bentler, 1998), and finally, the Akaike Information Criterion (AIC) indicated that the most optimal model is the one which generated the lowest value (Burnham and Anderson, 1998).

### Results

#### Exploratory Factor Analysis

The eigenvalue > 1 rule (Kaiser, 1960), supported by Cattell’s scree test (Cattell, 1966) suggested to consider three factors. Velicer’s Minimum Average Partial (MAP) test revealed 3 factors structure. However the revised version of the test suggested bifactorial structure (O’connor, 2000). Horn’s parallel test with Monte Carlo correction for principal axis analysis and row data permutation for a non-normally distributed data suggested a 3 factors structure, corroborating the result of the classic Velicer’s MAP test. These three factors explained 62.03% of the variance (48.87, 11.04, and 7.12%, respectively) (**Table 1**). We used a cut-off of 0.40 (Hair et al., 2006) to assess the practical significance of standardized factor loadings, but all the items loaded over 0.60 (**Table 1**). Items 4, 8, 9, 13, and 14 were problematic because they loaded approximately equally on factors 1 and 3. These cross-loaded items will be addressed in the discussion section of this study and examined in the following confirmatory factor analysis.

**Table 1:** Study 1 – Exploratory factor analysis of the 15 items of the PTQ with a principal axis factoring extraction method and Promax rotation.

	Factor 1	Factor 2	Factor 3
% of variance explained	48.87	11.04	7.12
(1) The same thoughts keep going through my mind again and again.	**0.734**	0.456	0.447
(2) Thoughts intrude into my mind.	0.531	0.335	**0.800**
(3) I can’t stop dwelling on them.	**0.847**	0.521	0.537
(4) I think about many problems without solving any of them.	0.674	0.655	0.329
(5) I can’t do anything else while thinking about my problems.	0.485	**0.754**	0.239
(6) My thoughts repeat themselves.	**0.790**	0.556	0.517
(7) Thoughts come to my mind without me wanting them to.	0.481	0.403	**0.838**
(8) I get stuck on certain issues and can’t move on.	0.672	0.760	0.373
(9) I keep asking myself questions without finding an answer.	0.639	0.660	0.349
(10) My thoughts prevent me from focusing other things.	0.560	**0.794**	0.379
(11) I keep thinking about the same issue all the time.	**0.809**	0.687	0.414
(12) Thoughts just pop into my mind.	0.507	0.401	**0.880**
(13) I feel driven to continue dwelling on the same issue.	0.711	0.657	0.467
(14) My thoughts are not much help to me.	0.495	0.461	0.237
(15) My thoughts take up all my attention.	0.457	**0.698**	0.390

*No item has been deleted in the table. Items 4, 8, 9, 13, and 14 cross-loaded on both factors 1 and 2. The values of items retained in respective factors are marked in bold.*

#### Confirmatory Factor Analysis

**FIGURE 1:**
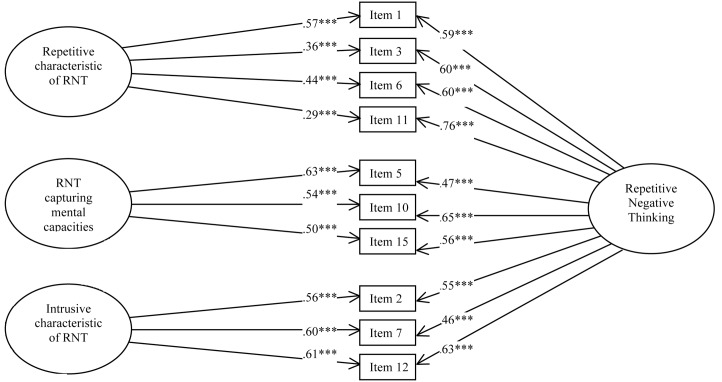
Study 1 – Path Diagram depicting the bifactor solution (Model C) of the French version of the PTQ. *^∗∗∗^p* < 0.001.

All goodness-of-fit indices indicated that model C (**Figure 1**) fits best the data (**Table 2**).

**Table 2:** Study 1 – Fit Index Value for the Different Tested Models.

Model	df	χ^2^	χ^2^/df	GFI	AGFI	RMSEA [90% CI]	RMR	CFI	AIC
A	32	67.09^∗∗∗^	2.10	0.96	0.94	0.06 [0.04; 0.08]	0.03	0.98	225.54
B	32	67.09^∗∗∗^	2.10	0.96	0.94	0.06 [0.04; 0.08]	0.03	0.98	538.23
**C**	**25**	**42.78^∗∗∗^**	**1.71**	**0.98**	**0.95**	**0.04 [0.02; 0.07]**	**0.02**	**0.99**	**102.78**
D	87	495.04^∗∗∗^	5.69	0.83	0.76	0.11 [0.10; 0.12]	0.06	0.86	561.04

*^∗∗∗^*p* < 0.001; Model A, a correlational solution with three subfactors; Model B, a hierarchical solution with one higher order factor and three subfactors; Model C, a bifactor solution with one common factor and three subfactors; Model D, a hierarchical solution with one higher order factor and three subfactors as validated by Ehring et al. (2011); *df*, degrees of freedom; χ^*2*^, Chi-Square; GFI, Goodness-of-fit Index; AGFI, Adjusted Goodness-of-fit Index; RMSEA, Root Mean Square Error of Approximation; RMSEA [90% CI], 90% Confidence Interval for RMSEA; RMR, Root Mean Square Residual; CFI, Comparative Fit Index; AIC, Akaike Information Criterion.*

Bifactor statistical indices were calculated, including omega, omega hierarchical and explained common variance (Rodriguez et al., 2016). The omega for the total score was 0.98 meaning that 98% of the variance was due to the factors and 2% was due to error. The omegas for the subfactors were 0.99 for the first factor, 0.98 for the second factor and 0.98 for the third factor. The omega hierarchical for the RNT was 0.81. Comparison of omega (0.98) and omega hierarchical (0.81) suggested that 83% of the reliable variance of the total score was attributable to the common factor RNT (0.81/0.98 = 0.83) and 17% was attributable to the subfactors (0.17/0.98 = 0.17). The omega hierarchicals for the subfactors were 0.07 for the first factor, 0.07 for the second factor and 0.05 for the third factor. Finally, explained common variance (ECV) was 0.82, suggesting that 82% of the common variance was attributable to the common factor RNT and 18% was spread among the three subfactors.

#### Internal Consistency

In the first sample, excellent internal consistencies were found (PTQ–total score, α = 0.89; factor 1, α = 0.88; factor 2, α = 0.88; factor 3, α = 0.82). The internal consistency was in the same range in the second sample (PTQ–total score, α = 0.88; factor 1, α = 0.84; factor 2, α = 0.84; factor 3, α = 0.83).

#### Convergent Validity

Spearman correlations were used because the data were non-normally distributed. The procedure of Benjamini and Hochberg (1995) was applied to detect the false discovery rate at 5% for the correlations. In the first sample, the PTQ–total score demonstrated significant positive correlations with other measures of RNT: the RRS–total score, the RRS–Reflection subscale, the RRS–Brooding subscale and the PSWQ (**Table 3**). Similarly, the three PTQ subscales showed significant correlations with the RRS–total score, the RRS–Reflection subscale, the RRS–Brooding subscale, and the PSWQ (**Table 3**).

**Table 3:** Study 1 – Intercorrelations between PTQ, other measures of RNT and anxiety in the first community sample (*N* = 364).

	1	2	3	4	5	6	7	8	9
(1) STAI-YB	–								
(2) PSWQ	0.73^∗∗∗^	–							
(3) RRS–Reflection	0.25^∗∗∗^	0.23^∗∗∗^	–						
(4) RRS–Brooding	0.61^∗∗∗^	0.59^∗∗∗^	0.37^∗∗∗^	–					
(5) RRS–Total	0.49^∗∗∗^	0.47^∗∗∗^	0.83^∗∗∗^	0.81^∗∗∗^	–				
(6) PTQ–Repetitive	0.64^∗∗∗^	0.59^∗∗∗^	0.28^∗∗∗^	0.54^∗∗∗^	0.48^∗∗∗^	–			
(7) PTQ–Mental Resources	0.49^∗∗∗^	0.45^∗∗∗^	0.32^∗∗∗^	0.40^∗∗∗^	0.44^∗∗∗^	0.56^∗∗∗^	–		
(8) PTQ–Intrusive	0.35^∗∗∗^	0.37^∗∗∗^	0.25^∗∗∗^	0.36^∗∗∗^	0.36^∗∗∗^	0.54^∗∗∗^	0.34^∗∗∗^	–	
(9) PTQ–Total	0.64^∗∗∗^	0.59^∗∗∗^	0.34^∗∗∗^	0.54^∗∗∗^	0.52^∗∗∗^	0.92^∗∗∗^	0.72^∗∗∗^	0.73^∗∗∗^	–

*^∗∗∗^p < 0.001; STAI-YB, the State Trait Anxiety Inventory-Trait; PSWQ, the Penn State Worry Questionnaire; RRS–Reflection, the reflection subscale of the Ruminative Response Scale-reconsidered; RRS–Brooding, the brooding subscale of the Ruminative Response Scale-reconsidered; RRS–Total, the total Ruminative Response Scale-reconsidered sum score; PTQ–Repetitive, the first factor of the Perseverative Thinking Questionnaire; PTQ–Intrusive, the second factor of the Perseverative Thinking Questionnaire; PTQ–Mental Resources, the third factor of the Perseverative Thinking Questionnaire; PTQ–Total, The total Perseverative Thinking Questionnaire sum score.*

To compare the Spearman *r*-values and determine whether correlations between the PTQ and subscales of RNT were different, Fisher’s r-to-*z* transformation and the Meng test of two correlations with one variable in common from the same sample were used (Meng et al., 1992). These analyses revealed that the correlation between the PTQ–total score and the RRS–Brooding subscale was significantly higher than between the PTQ–total score and the RRS–Reflection subscale (0.54 vs. 0.34, *p* < 0.05). Similarly, the correlations between the RRS–Brooding subscale and the PTQ–subscales 1 and 2 were significantly higher than between the RRS–Reflection subscale and subscales 1 and 2 of the PTQ (respectively, 0.54 vs. 0.28, *p* < 0.05 and 0.40 vs. 0.32, *p* < 0.05). Only the third subscale of the PTQ was equally correlated with the two subscales of RRS (*p* = 0.07, *ns*).

Consistently, Spearman correlations in the second community sample revealed that the PTQ–total score correlated significantly with other measures of RNT. Moreover, the three PTQ subscales showed significant positive correlations with the RRS–total score, the RRS–Reflection subscale, the RRS–Brooding subscale, and the PSWQ (**Table 4**).

**Table 4:** Study 1 – Intercorrelations between PTQ, other measures of RNT and measures of depression and anxiety in the second community sample (*N* = 361).

	1	2	3	4	5	6	7	8	9	10
(1.) STAI-YB	–									
(2.) CES-D	0.60^∗∗∗^	–								
(3) PSWQ	0.73^∗∗∗^	0.42^∗∗∗^	–							
(4) RRS–Reflection	0.21^∗∗∗^	0.29^∗∗∗^	0.21^∗∗∗^	–						
(5) RRS–Brooding	0.54^∗∗∗^	0.47^∗∗∗^	0.45^∗∗∗^	0.32^∗∗∗^	–					
(6) RRS–Tot	0.46^∗∗∗^	0.46^∗∗∗^	0.41^∗∗∗^	0.81^∗∗∗^	0.78^∗∗∗^	–				
(7) PTQ–Repetitive	0.58^∗∗∗^	0.43^∗∗∗^	0.50^∗∗∗^	0.19^∗∗∗^	0.44^∗∗∗^	0.39^∗∗∗^	–			
(8) PTQ–Mental resources	0.48^∗∗∗^	0.37^∗∗∗^	0.40^∗∗∗^	0.24^∗∗∗^	0.29^∗∗∗^	0.33^∗∗∗^	0.50^∗∗∗^	–		
(9) PTQ–Intrusive	0.43^∗∗∗^	0.30^∗∗∗^	0.35^∗∗∗^	0.22^∗∗∗^	0.33^∗∗∗^	0.34^∗∗∗^	0.56^∗∗∗^	0.36^∗∗∗^	–	
(10) PTQ–Total	0.63^∗∗∗^	0.46^∗∗∗^	0.52^∗∗∗^	0.26^∗∗∗^	0.43^∗∗∗^	0.43^∗∗∗^	0.91^∗∗∗^	0.73^∗∗∗^	0.73^∗∗∗^	–

*^∗∗∗^p < 0.001; STAI-YB, the State Trait Anxiety Inventory-Trait; CES-D, The Center for Epidemiologic Studies Depression Scale Revised; PSWQ, the Penn State Worry Questionnaire; RRS–Reflection, the reflection subscale of the Ruminative Response Scale-reconsidered; RRS–Brooding, the brooding subscale of the Ruminative Response Scale-reconsidered; RRS–Total, the total Ruminative Response Scale-reconsidered sum score; PTQ–Repetitive, the first factor of the Perseverative Thinking Questionnaire; PTQ–Intrusive, the second factor of the Perseverative Thinking Questionnaire; PTQ–Mental Resources, the third factor of the Perseverative Thinking Questionnaire; PTQ–Total, The total Perseverative Thinking Questionnaire sum score. CES-D, The Center for Epidemiologic Studies Depression Scale Revised.*

Fisher’s r-to-*z* transformation and the Meng test revealed that the correlation between the PTQ–total score and the RRS–Brooding subscale was significantly higher than between the PTQ–total score and the RRS–Reflection subscale (0.43 vs. 0.26, *p* < 0.05). Moreover, the correlations between the RRS–Brooding subscale and the PTQ–subscale 1 and the PTQ–subscale 2 were significantly higher than between the RRS–Reflection subscale and the PTQ–subscale 1 and the PTQ–subscale 3 (respectively, 0.44 vs. 0.19, *p* < 0.05 and 0.33 vs. 0.22, *p* < 0.05). Finally, PTQ-subscale 2 was equally correlated with the two subscales of RRS (*z* = 0.20, *ns*).

#### Predictive Validity

Regression analysis revealed that the PTQ–total score among both first and second community sample predicted significantly anxiety and depression symptoms. The PTQ–total score accounted for 42% of the variance in anxiety symptoms among the first community sample, for 40% of the variance in anxiety symptoms and for 21% of the variance in depression symptoms among the second community sample. Hierarchical regressions were conducted to examine the independent contributions of the subscales to predict anxiety and depression. Among the first community sample, the PTQ subscale accounted for 46% of the variance in anxiety symptoms (**Table 5**) whereas, among the second community sample, the PTQ subscales accounted for 41% of the variance in anxiety symptoms and 22% of the variance in depression symptoms (**Table 6**). Moreover, subscales 1 and 2 significantly predicted anxiety and depression.

**Table 5:** Study 1 – Hierarchical regression analysis of PTQ subfactors on anxiety in the first community sample (*N* = 361).

	Anxiety *R* = 0.68, *R*^2^ = 0.46			
Subfactors	*β*	*B*	*SE B*	*95%* CI
PTQ–Repetitive	0.54^∗∗∗^	1.62	0.17	[1.28, 1.96]
PTQ–Mental Resources	0.21^∗∗∗^	0.83	0.20	[0.44, 1.23]
PTQ–Intrusive	-0.01	-0.04	0.20	[-0.43, 0.34]

*^∗∗∗^p < 0.001; PTQ–Repetitive, the first factor of the Perseverative Thinking Questionnaire; PTQ–Mental Resources, the second factor of the Perseverative Thinking Questionnaire; PTQ–Intrusive, the third factor of the Perseverative Thinking Questionnaire.*

**Table 6:** Study 1 – Hierarchical regression analysis of PTQ subfactors on anxiety and depression in the second community sample (*N* = 361).

	Anxiety *R* = 0.64, *R*^2^ = 0.41				Depression *R* = 0.47, *R*^2^ = 0.22			
Subfactors	*β*	*B*	*SE B*	*95%* CI	*β*	*B*	*SE B*	*95%* CI
PTQ–Repetitive	0.41^∗∗∗^	1.32	0.19	[0.96, 1.68]	0.32^∗∗∗^	0.68	0.14	[0.39, 0.96]
PTQ–Mental Resources	0.26^∗∗∗^	0.95	0.18	[0.59, 1.31]	0.21^∗∗∗^	0.51	0.14	[0.23, 0.79]
PTQ–Intrusive	0.09	0.36	0.22	[-0.07, 0.79]	0.03	0.07	0.17	[-0.27, 0.40]

*^∗∗∗^p < 0.001; PTQ–Repetitive, the first factor of the Perseverative Thinking Questionnaire; PTQ–Mental Resources, the second factor of the Perseverative Thinking Questionnaire; PTQ–Intrusive, the third factor of the Perseverative Thinking Questionnaire.*

### Discussion

The aim of the first study was to explore and confirm the factorial structure and psychometric properties of the French version of the PTQ in general population. The exploratory factor analysis revealed a three-factor structure as in the original version but with a different items organization in each factor. The first factor, which measures the difficulties of disengaging from RNT and the repetitiveness of RNT, is composed of four items. The second factor, which assesses the capture of mental resources by RNT, is composed of three items. The third factor, which assesses the intrusiveness of RNT, is also composed of three items. Items 4, 8, 9, 13, and 14 from the original version of the PTQ were problematic because they were cross-loading on the first and the second factor in the French version of the questionnaire. The meaning of items 4, 9, and 14 corresponded to the unproductiveness characteristic of RNT. This feature was included in the work definition of RNT developed by Ehring et al. (2011). Nevertheless, several studies demonstrated that individuals perceive RNT as an adaptive strategy to cope with negative mood and develop positive beliefs about the use of RNT (Lyubomirsky and Nolen-Hoeksema, 1993; Papageorgiou and Wells, 2001; Watkins and Baracaia, 2001). The perception of the usefulness aspect of RNT by individuals remains unresolved and need to be addressed in further studies. Moreover, item 8 was also cross-loading on factors one and two. It is possible that this result is due to the two-part formulation of the item: the sense of the beginning of the item 8 (“I get stuck on certain issues”) may correspond to the first factor (i.e., repetitiveness of RNT), while the end (“and can’t move on”) could load on the second factor (i.e., capture of mental resources). Therefore, item 8 was deleted. Finally, the meaning of item 13 fitted better with factor one and the difficulties of disengaging from RNT but because it was cross-loaded on factor one and two, we decided to deleted it from the final version, producing a 10-item questionnaire.

Confirmatory factor analysis conducted on another non-clinical sample confirmed the factorial structure revealed in the first sample and suggested that a bifactor model composed of 10 items with the RNT as a common factor and the three subfactors fits better with our data. The item composition of factors one and three differed from Ehring et al. (2011)’s original version. Only the second factor was exactly the same.

The validity of the French version of the PTQ in the general population was confirmed through excellent internal consistencies of the PTQ–total score and each PTQ factors in the two independent community samples. Convergent validity was demonstrated through positive correlations between the PTQ–total score, the three subscale scores and other measures of RNT. Moreover, the association between the PTQ and the brooding factor was significantly higher than the link between the PTQ and the reflection factor. According to Treynor et al. (2003), the brooding factor of rumination is a passive comparison of one’s current situation with some unachieved standards, reflecting a maladaptive process of rumination that is associated with more negative consequences than the reflection factor. This latter factor refers to a purposeful turning inward to engage in cognitive problem solving to alleviate one’s depressive symptoms and is associated with more adaptive coping strategies. Consequently, the PTQ appears to be a good scale to assess maladaptive repetitive thinking. This point is underlined by the predictive validity established with significant regression analysis between the PTQ and the measure of anxiety and depression symptoms, confirming the capacity of the PTQ to detect RNT and to predict the associated negative mood. Surprisingly, the third subscale of the PTQ (i.e., Intrusiveness) did not predict significantly anxiety and depression symptoms. Moreover, because the explained common variance (ECV) suggested that 82% of the common variance was attributable to the common factor RNT, it seems more appropriate to use the PTQ total score to assess RNT. To conclude, the French version of the PTQ is a good scale to assess independent-content RNT in a general population using the PTQ total score. The three sub-scores can serve to examine specific features of RNT to provide more detailed information but seem less appropriate than the total score of RNT.

## Study 2

The second study aimed to validate the French version of the PTQ in a clinical population through a confirmatory factor analysis and to examine the psychometric properties in a sample including different clinical populations. The Beck Depression Inventory replaced the CES-D to assess depressive symptoms because it is a validated and widely used questionnaire to examine depression in a clinical population.

### Method

#### Participants

Three hundred and eighteen participants were recruited in eight French mental health clinics. Participants with a primary diagnosis of alcohol dependence (*n* = 147), chronic pain disorder (*n* = 110), generalized anxiety disorder and major depressive disorder (*n* = 39) and eating disorder (*n* = 22) were selected due to the use of the RNT as a development and maintenance factor in these disorders (Ehring and Watkins, 2008; Nolen-Hoeksema and Watkins, 2011). Inclusion criteria were (1) being between 18 and 64 years old and (2) speaking and reading French fluently. Exclusion criteria were having a diagnosis established by a medical doctor of (1) a serious somatic problem, (2) serious cognitive deficits, or (3) a psychotic disorder. Specifically, for the alcohol dependent group, participants had to be diagnosed with alcohol dependence by a medical doctor according to the criteria of the International Statistical Classification of Diseases and Related Health Problems (ICD-10) (World Health Organization, 2000). They had no substance use in the last 15 days (except for tobacco) and did not have other dependence issues (except for tobacco). For the chronic pain disorder group, participants were diagnosed by a medical doctor according to the definition of chronic pain by the International Association for the Study of Pain (Task Force on Taxonomie of the International Association for the Study of Pain, 2011). Participants of the anxious and depressive group were diagnosed with generalized anxiety disorder or major depressive disorder by a medical doctor according to the fifth version of the Diagnostic and Statistical Manual of Mental Disorders (DSM-5) (American Psychiatric Association, 2013). Finally, participants from the eating disorder group were diagnosed by a medical doctor with bulimia nervosa without compensation behavior or with eating disorder not otherwise specified according to the DSM-5 criteria (American Psychiatric Association, 2013). Patients meeting the inclusion criteria were approached by the experimenter. All participants read and signed the information letter and gave their written informed consent. The participants completed a paper-and-pencil version of the questionnaires. No personal data allowing personal identification were requested. Participants with missing data and univariate and multivariate outliers were removed. Participants of the final sample (*N* = 291; female: 151; age: *M* = 46.17, *SD* = 10.17) suffered from alcohol dependence (48%), chronic pain (30%), major depressive disorder (10%), anxiety disorder (4%) and eating disorder (8%). The study protocol was conducted with the approval of the Ethical Committee in Behavioral Science of the University of Lille (France) (ref. 2014-2-S23) and carried out according to the recommendations of the American Psychological Association and the 1964 Declaration of Helsinki.

#### Measures

##### The Perseverative Thinking Questionnaire

The French version of *The Perseverative Thinking Questionnaire* used in study 1 was also used in the second study.

##### Other measures of RNT

*The Ruminative Response Scale-reconsidered* (Treynor et al., 2003; Baeyens et al., in preparation) and *The Penn State Worry Questionnaire* (Meyer et al., 1990; Gosselin et al., 2001) were also used in the second study to establish convergent validity. Internal consistencies were acceptable for both factors of rumination (for total score, α = 0.75; for brooding, α = 0.67; for reflection, α = 0.66) and very good for the PSWQ (α = 0.89). *The Mini Cambridge Exeter Ruminative Thought Scale* (Mini-CERTS; Douilliez et al., 2014) was added to evaluate two distinct modes of thoughts according to the Processing Mode Theory (Watkins, 2004, 2008). This self-report questionnaire consists of eight items evaluating the quantity of abstract-analytic thinking (AAT), an unconstructive form of passive analysis of the causes, consequences and meanings of an event (e.g., item 1: “My thinking tends to get stuck in a rut, involving only a few themes”) and 7 items evaluating concrete-experiential thinking (CET), a constructive form of thought implying an attentional focalization on the present moment, one’s feelings, physiological sensations and environmental details (e.g., item 2: “I can grasp and respond to changes in the world around me without having to analyze the details”). Experimental literature has demonstrated that, compared to a concrete–experiential mode of thinking, an abstract–analytic mode of thinking leads to an increase in negative mood (Watkins, 2004; Moberly and Watkins, 2006; Watkins et al., 2009) and impaired problem resolution (Watkins and Moulds, 2005). The abstract-analytic subscale was used to evaluate convergent validity, and the concrete–experiential subscale was used to examine divergent validity. Participants were instructed to rate the items to reflect how they typically think when they are confronted with a difficult situation. They responded using a 4-point Likert scale ranging from 1 (*almost never*) to 4 (*almost always*). A higher score on each dimension reflects a high level of the type of repetitive thinking considered. This scale demonstrated an acceptable level of internal consistency (for CET, α = 0.77; for AAT, α = 0.75) and AAT was positively correlated with measures of depression, anxiety and rumination (Douilliez et al., 2014). In the current study, internal consistency was good (for CET, α = 0.71; for AAT, α = 0.79).

##### Depression and anxiety

*The State Trait Anxiety Inventory-Trait* (Spielberger, 1989; Gauthier and Bouchard, 1993) was used to assess recurrent anxiety, and internal consistency for the STAI-YB was excellent (α = 0.91). *The Beck Depression Inventory* (Beck et al., 1996, 1998) was used to assess depression in clinical samples. Internal consistency for the BDI-II was excellent (α = 0.91).

#### Data Analysis

Amos 23 (Arbuckle, 2014) was used to perform the confirmatory factor analysis, comparing the same four models as in the first study. Robust maximum likelihood estimation was used to examine the fit of the four models due to the non-normal distribution of our data (Mardial’s test of multivariate kurtosis = 20.82, *p* < 0.001; Small’s test of multivariate normality = 47.80, *p* < 0.001) (Mardia, 1975) and ordinality of the items. As in study 1, the goodness-of-fit indices considered were the normed chi-square (the chi-square on the number of degrees of freedom) which is acceptable lower than 3 (Schumacker and Lomax, 2004), the Goodness-of-Fit Index (GFI) and its corresponding adjusted version (AGFI) for which the minimum value for model acceptance is 0.80 (Cole, 1987), the Root Mean Square Error of Approximation (RMSEA) which is considered as good when lower than 0.05 and acceptable if lower than 0.10, and the Root Mean Square Residual (RMR) which must be as low as possible and considered as acceptable when lower than 0.05 (Cole, 1987), the Comparative Fit Index (CFI) comparing the model of interest with alternatives which have to exceed 0.90 (Hu and Bentler, 1998), and finally, the Akaike Information Criterion (AIC) indicated that the most optimal model is the one which generated the lowest value (Burnham and Anderson, 1998).

### Results

#### Confirmatory Factor Analysis

**FIGURE 2:**
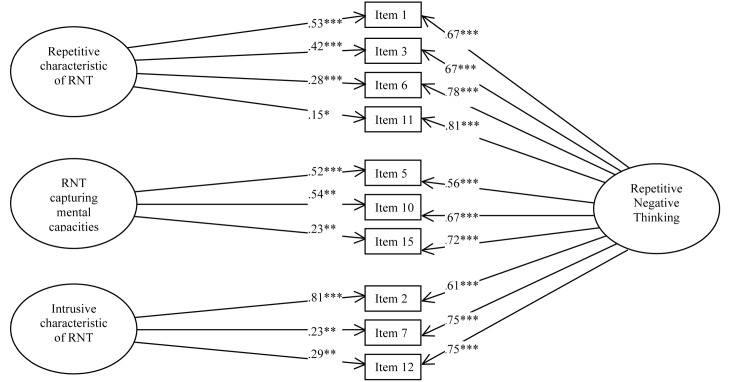
Study 2 – Path Diagram depicting the bifactor solution (Model C) of the French version of the PTQ. *^∗∗∗^p* < 0.001, *^∗∗^p* < 0.01, *^∗^p* < 0.05.

According to two previous studies, our data fit better with the bifactor model (model C) (see **Table 7** for the fit index and **Figure 2** for the path diagram). The omega for the total score was 0.98 meaning that 98% of the variance was due to the factors and 2% was due to error. The omegas for the subfactors were 0.98 for the first factor, 0.98 for the second factor and 0.98 for the third factor. The omega hierarchical for the RNT was 0.87. Comparison of omega (0.98) and omega hierarchical (0.87) suggested that 89% of the reliable variance of the total score was attributable to the common factor RNT (0.87/0.98 = 0.89) and 11% was attributable to the subfactors (0.11/0.98 = 0.11). The omega hierarchicals for the subfactors were 0.05 for the first factor, 0.02 for the second factor and 0.04 for the third factor. Finally, explained common variance (ECV) was 0.89, suggesting that 89% of the common variance was attributable to the common factor RNT and 11% was spread among the three subfactors.

**Table 7:** Study 2 – Fit Index Value for the Different Tested Models in the clinical sample (*N* = 291).

Model	df		χ^2^	χ^2^/df		GFI	AGFI	RMSEA [90% CI]	RMR	CFI	AIC
A	32		130.36^∗∗∗^	4.07		0.91	0.85	0.10 [0.08; 0.12]	0.06	0.95	176.36
B	32		130.36^∗∗∗^	4.07		0.91	0.85	0.10 [0.08; 0.12]	0.06	0.95	176.36
**C**	**25**		**61.87^∗∗∗^**	**2.47**		**0.96**	**0.91**	**0.07 [0.05; 0.09]**	**0.04**	**0.98**	**121.87**
D	87		360.83^∗∗∗^	4.15		0.85	0.79	0.10 [0.09; 0.12]	0.06	0.90	426.83

*^∗∗∗^p < 0.001; Model A, a correlational solution with three subfactors; Model B, a hierarchical solution with one higher order factor and three subfactors; Model C, a bifactor solution with one common factor and three subfactors; Model D, a hierarchical solution with one higher order factor and three subfactors as validated by Ehring et al. (2011); df, degrees of freedom; χ^2^, Chi-Square; GFI, Goodness-of-fit Index; AGFI, Adjusted Goodness-of-fit Index; RMSEA, Root Mean Square Error of Approximation; RMSEA [90% CI], 90% Confidence Interval for RMSEA; RMR, Root Mean Square Residual; CFI, Comparative Fit Index; AIC, Akaike Information Criterion.*

#### Internal Consistency

Excellent internal consistencies were found for the PTQ–total score (α = 0.92) as well as for the factor 1 (α = 0.88), the factor 2 (α = 0.82), and the factor 3 (α = 0.84).

#### Convergent Validity

The procedure of Benjamini and Hochberg (1995) did not detect a false discovery rate at 5% for the 66 correlations examined. Spearman correlations demonstrated significant positive correlations between the PTQ–total score and other measures of RNT. Moreover, the three PTQ subscales correlated significantly with other measures of RNT (**Table 8**).

**Table 8:** Study 2 – Intercorrelations between PTQ, other measures of RNT, measures of depression and anxiety in the clinical sample (*N* = 291).

	1	2	3	4	5	6	7	8	9	10	11	12
(1) STAI-YB	–											
(2) BDI-II	0.78^∗∗∗^	–										
(3) PSWQ	0.71^∗∗∗^	0.57^∗∗∗^	–									
(4) RRS–Reflection	0.26^∗∗^	0.27^∗∗∗^	0.29^∗∗∗^	–								
(5) RRS–Brooding	0.61^∗∗∗^	0.62^∗∗∗^	0.54^∗∗∗^	0.44^∗∗∗^	–							
(6) RRS–Tot	0.52^∗∗∗^	0.53^∗∗∗^	0.49^∗∗∗^	0.82^∗∗∗^	0.86^∗∗∗^	–						
(7) MINI CERTS-AAT	0.72^∗∗∗^	0.68^∗∗∗^	0.66^∗∗∗^	0.33^∗∗∗^	0.62^∗∗∗^	0.57^∗∗∗^	–					
(8) MINI CERTS-CET	-0.29^∗∗∗^	-0.26^∗∗^	-0.22^∗∗^	0.19^∗^	-0.05	0.08	-0.10	–				
(9) PTQ–Repetitive	0.62^∗∗∗^	0.60^∗∗∗^	0.58^∗∗∗^	0.28^∗∗^	0.49^∗∗∗^	0.45^∗∗∗^	0.59^∗∗∗^	-0.16^∗^	–			
(10) PTQ– Mental Resources	0.62^∗∗∗^	0.62^∗∗∗^	0.52^∗∗∗^	0.23^∗∗^	0.47^∗∗∗^	0.42^∗∗∗^	0.57^∗∗∗^	-0.19^∗∗^	0.66^∗∗∗^	–		
(11) PTQ–Intrusive	0.60^∗∗∗^	0.54^∗∗∗^	0.62^∗∗∗^	0.30^∗∗∗^	0.46^∗∗∗^	0.44^∗∗∗^	0.52^∗∗∗^	-0.14	0.78^∗∗∗^	0.58^∗∗∗^	–	
(12) PTQ–Total	0.67^∗∗∗^	0.64^∗∗∗^	0.62^∗∗∗^	0.30^∗∗∗^	0.52^∗∗∗^	0.48^∗∗∗^	0.62^∗∗∗^	-0.18^∗∗^	0.95^∗∗∗^	0.80^∗∗∗^	0.87^∗∗∗^	–

*^∗^p < 0.05; ^∗∗^p < 0.001; ^∗∗∗^p < 0.01; STAI-YB, the State Trait Anxiety Inventory-Trait; BDI-II, The Beck Depression Inventory II; PSWQ, the Penn State Worry Questionnaire; RRS–Reflection, the reflection subscale of the Ruminative Response Scale-reconsidered; RRS–Brooding, the brooding subscale of the Ruminative Response Scale-reconsidered; RRS–Total, the total Ruminative Response Scale-reconsidered sum score; AAT, The Mini Cambridge Exeter Ruminative Thought Scale – Abstract–Analytic Thinking subscale; CET, The Mini Cambridge Exeter Ruminative Thought Scale – Concrete–Experiential Thinking subscale; PTQ–Repetitive, the first factor of the Perseverative Thinking Questionnaire; PTQ–Mental Resources, the second factor of the Perseverative Thinking Questionnaire; PTQ–Intrusive, the third factor of the Perseverative Thinking Questionnaire; PTQ–Total, The total Perseverative Thinking Questionnaire sum score.*

Fisher’s r-to-*z* transformation and the Meng test revealed that the correlation between the PTQ–total score and the RRS-Brooding subscale was significantly higher than between the PTQ–total score and the RRS-Reflection subscale (0.52 vs. 0.30, *p* < 0.05). The correlations between PTQ–factor 1 and RRS-Brooding subscale were significantly higher than the correlation between PTQ–factor 1 and RRS-Reflection subscale (0.58 vs. 0.28, *p* < 0.05). The correlation between PTQ–factor 2 and the RRS-Brooding subscale was significantly higher than the correlation between PTQ–factor 2 and RRS-Reflection subscale (0.47 vs. 0.23, *p* < 0.05). The correlation between the PTQ–factor 3 and RRS-Brooding subscale was significantly higher than the correlation between the PTQ–factor 3 and RRS-Reflection subscale (0.46 vs. 0.30, *p* < 0.05).

#### Divergent Validity

Negative correlations between the Mini CERTS Concrete–Experiential thinking (CET) subscale and the PTQ–total score, PTQ–factor 1 and PTQ–factor 2 were significant (**Table 8**). Interestingly, the only positive significant correlation with the Mini CERTS–CET subscale was the RRS–Reflection subscale (*r* = 0.19, *p* < 0.05).

#### Predictive Validity

Regression analysis revealed that the PTQ–total score among clinical sample predicted significantly anxiety and depressive symptoms. The PTQ–total score accounted for 49% of the variance in anxiety symptoms and for 38% of the variance in depression symptoms. Hierarchical regressions were conducted to examine the independent contributions of the subscales to predict anxiety and depression. The PTQ subscales accounted for 53% of the variance in anxiety symptoms and 41% of the variance in depression symptoms (**Table 9**). Moreover, subscales 1 and 2 predicted significantly anxiety, but only subscale 2 predicted significantly anxiety and depression symptoms (*p* < 0.001).

**Table 9:** Study 2 – Hierarchical regression analysis of PTQ subfactors on anxiety and depression in the clinical sample (*N* = 291).

	Anxiety *R* = 0.73, *R*^2^ = 0.53				Depression *R* = 0.64, *R*^2^ = 0.41			
Subfactors	*β*	*B*	*SE B*	*95%* CI	*β*	*B*	*SE B*	*95%* CI
PTQ–Repetitive	0.25^∗^	0.74	0.29	[0.17, 1.30]	0.19	0.68	0.48	[-0.27, 1.63]
PTQ–Mental Resources	0.38^∗∗∗^	1.44	0.25	[0.94, 1.94]	0.41^∗∗∗^	1.91	0.43	[1.06, 2.76]
PTQ–Intrusive	0.17	0.70	0.36	[-0.01, 1.40]	0.10	0.49	0.61	[-0.73, 1.69]

*^∗^p < 0.05, ^∗∗∗^p < 0.001; PTQ–Repetitive, the first factor of the Perseverative Thinking Questionnaire; PTQ–Mental Resources, the second factor of the Perseverative Thinking Questionnaire; PTQ–Intrusive, the third factor of the Perseverative Thinking Questionnaire.*

### Discussion

Study 2 confirmed that the bifactor model composed of 10 items fit better with our data. The items were distributed in one common factor and three subfactors. Internal consistencies of the PTQ total score and of the three PTQ subscales were excellent. Correlations between the PTQ and other measures of RNT confirmed the convergent validity of the tool. The divergent validity was established with the negative significant link between the PTQ and Abstract–analytic thinking. Finally, the PTQ–total score predicted significantly anxiety and depression. Nevertheless, because the PTQ–Intrusiveness subscale did not significantly predict anxiety and depression symptoms and moreover, because the explained common variance suggested that 89% of the common variance was attributable to the common factor RNT, the current study confirms that the total score of the French PTQ is more appropriate to assess RNT rather than three subscores.

If current analyses demonstrated that the PTQ provides a valid measure of independent-content RNT in clinical populations, it is necessary to address some limits. Firstly, diagnoses of participants were based on a clinical interview. No structured clinical interview was conduct to control the primary diagnosis and identify comorbid disorder. In addition, the analyzes carried out on a sample mixing all the disorders does not allow to identify the tendency to have RNT within each diagnosis. Finally, our mixed clinical sample is mainly composed participants suffering from alcohol dependence disorder and chronic pain, limiting generalizability of our results to other emotional disorders. Further studies need to address this limitation by gathering more data in population suffering from depression and anxiety disorder.

## General Discussion

The aim of the two current studies was to translate and validate the French version of the PTQ (Ehring et al., 2011) in both general and clinical populations. The adaptation of a psychological instrument in other languages and other cultures requires strong methodological rigor (Borsa et al., 2012). It is important that the adapted scale reflects the same content, psychometric properties and general validity for the individuals concerned. To ensure the semantic equivalence of items, we followed the steps for the trans-cultural validation of psychometric instruments (Hambleton et al., 2006). Then, psychometric properties have been verified through rigorous statistical analyzes (i.e., factorial structure, internal consistency, convergent validity, predictive validity and divergent validity) and comparing with scales assessing different types of RNT (i.e., rumination, worry), different modes of RNT (i.e., abstract-analytic thinking and concrete-experiential thinking) and different symptomatologies (i.e., anxiety and depression). The exploratory factor analysis conducted in the first study revealed a three factors composition with 10 items. It suggested that items 4, 8, 9, 13, and 14 of the original German version were cross-loaded on the first and the second factor in the French version. Finally, the French version of the PTQ was completed by three separate samples with two independent sample of individuals from the general population and one sample with individual from clinical population. The multiplication of the validation studies in separate samples increase the scale validity. The confirmatory factor analysis conducted in the second community sample and in the clinical sample in Study 2 demonstrated that the best model was a bifactor model with RNT as common factor and three subfactors: the first factor, measuring the difficulties of disengaging from RNT and the repetitiveness of RNT, is composed of four items (items 1, 3, 6, and 11); the second factor, assessing the capture of mental resources by RNT, is composed of three items (items 5, 10, and 15); and the third factor, which assesses the intrusiveness of RNT, is composed of three items (items 2, 7, and 12). The second factor assessing mental resources captured by RNT was the same as in the original version, but factors 1 and 3 were slightly different, and five items were deleted.

The internal consistencies of the PTQ–total score and of each PTQ factor were excellent and confirmed the validity of the French version of the PTQ in the general and the clinical populations. The correlations revealed that the French PTQ was linked with other measures of unconstructive RNT (RRS-brooding, PSWQ and Mini-CERTS-AAT), confirming the convergent validity of the scale. Interestingly, correlations between the PTQ and the brooding factor of rumination, assessed with the RRS, were higher than correlations between the PTQ and the reflection subscale of rumination from the RRS. These results indicated that reflection is a more adaptive form of rumination than brooding (Treynor et al., 2003). The divergent validity was shown through a negative correlation between the PTQ and the concrete–experiential mode of thinking, a mode of thinking associated with better problem resolution and with a decrease of negative mood (Watkins, 2004; Moberly and Watkins, 2006). These interesting results support the Processing Mode Theory (Watkins, 2004, 2008) suggesting that RNT were processed on an abstract–analytic mode and that concrete–experiential mode is a more adaptive form of repetitive thinking. The PTQ total score predicted significantly measures of anxiety and depression supporting the transdiagnostic role of the RNT assessed by the PTQ. Nevertheless, it should be outlined that the second subscale was not a significant predictor of anxiety and depression symptoms. Moreover, the explained common variance suggested that 89% of the common variance was attributable to the common factor RNT. These indices suggest to use the total score of the French PTQ to assess RNT, rather than the three subscores which can still be used to understand some important features of RNT.

It is important to note that we did not examine the retest reliability and further studies will have to establish the longitudinal validity of the French PTQ. Moreover, we must note that the assumption that individuals perceive the unproductive aspect of RNT has not been consistently observed. For instance, Michael et al. (2007) showed a link between psychopathology and self-reported unproductiveness of RNT in individuals suffering from post-traumatic stress disorder, however, other studies have found that patients suffering from other disorders considered repetitive thinking a productive coping strategy. Repetitive thinking can be subjectively perceived by patients as an adaptive strategy to cope with negative mood by finding solutions that might ultimately resolve the patients’ problems or by preparing themselves for the worst (Lyubomirsky and Nolen-Hoeksema, 1993; Papageorgiou and Wells, 2001; Watkins and Baracaia, 2001). This RNT characteristic should be studied in the future to determine whether individuals generally perceive repetitive thinking as a productive or unproductive coping strategy. This characteristic is fundamental to improve understanding of the process and its treatment. The answer might be found in the work of Papageorgiou and Wells (2001), which demonstrated that rumination is linked to positive beliefs and considered rumination a coping strategy (e.g., “Ruminating about the past helps me to prevent future mistakes and failures”). This key characteristic of RNT remains unaddressed, although it seems to be central in understanding the inclination to ruminate.

## Conclusion

The PTQ is a valid scale to assess RNT in French speakers from both the general and clinical populations. The transdiagnostic perspective of the PTQ adds value to existing tools that are disorder-specific and might be useful in research as well as in clinical contexts. This content-independent scale measures RNT in individuals without specific disorders as well as in clinical population. Considering that French is spoken in more than twenty-nine countries in the world (International Organization of Francophonie, 2014), the French version of the PTQ is the necessary starting point to develop researches about the role played by RNT in development and maintenance of differents disorders among individuals speaking French language. The French version of this scale will contribute to improve our understanding the role played by RNT among some disorders in the field of experimental psychopathology as it provides the first content-independent scale for French community.

## Author Contributions

FD: conception and design; analysis and interpretation of results; writing manuscript. MK: conception; analysis and interpretation of results; revising manuscript. CB: conception; analysis and interpretation of results; revising manuscript. ÉS, JF, BG, CD, PT, OD, PhT and FS: conception; acquisition of data; revising manuscript. AR: analysis and interpretation of results; revising manuscript. LR: conception; acquisition of data; analysis and interpretation of results; revising manuscript.

## Conflict of Interest Statement

The authors declare that the research was conducted in the absence of any commercial or financial relationships that could be construed as a potential conflict of interest.

## APPENDIX

**Table A:** The English version of the Perseverative Thinking Questionnaire.

	Never	Rarely	Sometimes	Often	Almost always
(1) The same thoughts keep going through my mind again and again.	0	1	2	3	4
(2) Thoughts intrude into my mind.	0	1	2	3	4
(3) I can’t stop dwelling on them.	0	1	2	3	4
(4) I think about many problems without solving any of them.	0	1	2	3	4
(5) I can’t do anything else while thinking about my problems.	0	1	2	3	4
(6) My thoughts repeat themselves.	0	1	2	3	4
(7) Thoughts come to my mind without me wanting them to.	0	1	2	3	4
(8) I get stuck on certain issues and can’t move on.	0	1	2	3	4
(9) I keep asking myself questions without finding an answer.	0	1	2	3	4
(10) My thoughts prevent me from focusing other things.	0	1	2	3	4
(11) I keep thinking about the same issue all the time.	0	1	2	3	4
(12) Thoughts just pop into my mind.	0	1	2	3	4
(13) I feel driven to continue dwelling on the same issue.	0	1	2	3	4
(14) My thoughts are not much help to me.	0	1	2	3	4
(15) My thoughts take up all my attention.	0	1	2	3	4

*Instruction: In this questionnaire, you will be asked to describe how you *typically* think about negative experiences and problems. Please read the following statements and rate the extent to which they apply to you when you think about negative experiences or problems*.

**Table B:** The French version of the Perseverative Thinking Questionnaire.

		Jamais	Rarement	Parfois	Souvent	Presque toujours
Item 1	(1) Les mêmes pensées continuent à traverser mon esprit encore et encore.	0	1	2	3	4
Item 2	(2) Des pensées s’immiscent dans mon esprit.	0	1	2	3	4
Item 3	(3) Je ne peux pas m’arrêter d’y penser.	0	1	2	3	4
Deleted	(4) Je pense à beaucoup de problèmes sans réussir à en résoudre un seul.	0	1	2	3	4
Item 4	(5) Je ne peux rien faire d’autre lorsque je suis en train de penser à mes problèmes.	0	1	2	3	4
Item 5	(6) Mes pensées se répètent.	0	1	2	3	4
Item 6	(7) Des pensées me viennent à l’esprit sans que je le veuille.	0	1	2	3	4
Deleted	(8) Je reste bloqué(e) sur certains problèmes et n’arrive pas à aller de l’avant.	0	1	2	3	4
Deleted	(9) Je continue à me poser des questions sans réussis à trouver de réponse.	0	1	2	3	4
Item 7	(10) Mes pensées m’empêchent de me concentrer sur d’autres choses.	0	1	2	3	4
Item 8	(11) Je continue à penser aux mêmes problèmes tout le temps.	0	1	2	3	4
Deleted	(12) Des pensées surgissent dans mon esprit.	0	1	2	3	4
Deleted	(13) Je me sens poussé(e) à continuellement penser aux mêmes problèmes.	0	1	2	3	4
Item 9	(14) Mes pensées ne sont pas d’une grande aide pour moi.	0	1	2	3	4
Item 10	(15) Mes pensées mobilisent toute mon attention.	0	1	2	3	4

*Consignes: Dans ce questionnaire, nous allons vous demander de décrire la manière dont vous pensez habituellement à des problèmes ou des expériences négatives. Lisez chacune des propositions présentées ci-dessous, et évaluez dans quelle mesure cela s’applique à vous lorsque vous pensez à des expériences négatives ou à des problèmes. Ne passez pas trop de temps à répondre, c’est votre première impression qui est importante*.
